# Supplementary material for: Adaptive Optics-Transscleral Flood Illumination Imaging of Retinal Pigment Epithelium in Dry Age-Related Macular Degeneration
Source: Cells. 2025 Apr 24;14(9):633. doi: 10.3390/cells14090633 (PMC12071642; doi:10.3390/cells14090633)

Figure S1. Results of the custom correlation plugin. Example in early AMD with subretinal drusenoid deposits (Temporal quadrant of left eye, Female, 72 years). **(a) Registration** of the mask of the low-resolution AO-TFI mosaic on IR fundus to identify the OCT B-scans corresponding to the AO-TFI mosaic. Example: image #141 over the full "AO-TFI-on-OCT-stack" of 193 images. **(b) Stack image 103 of the full "Correlation fundus-AO-TFI-OCT" dataset available in Movie S1.** The wrapped AO-TFI mosaic (center) is correlated with IR fundus image (left panel) cropped to fit the area imaged with AO-TFI. The white lines indicate the location of the OCT B-scan (right panel).

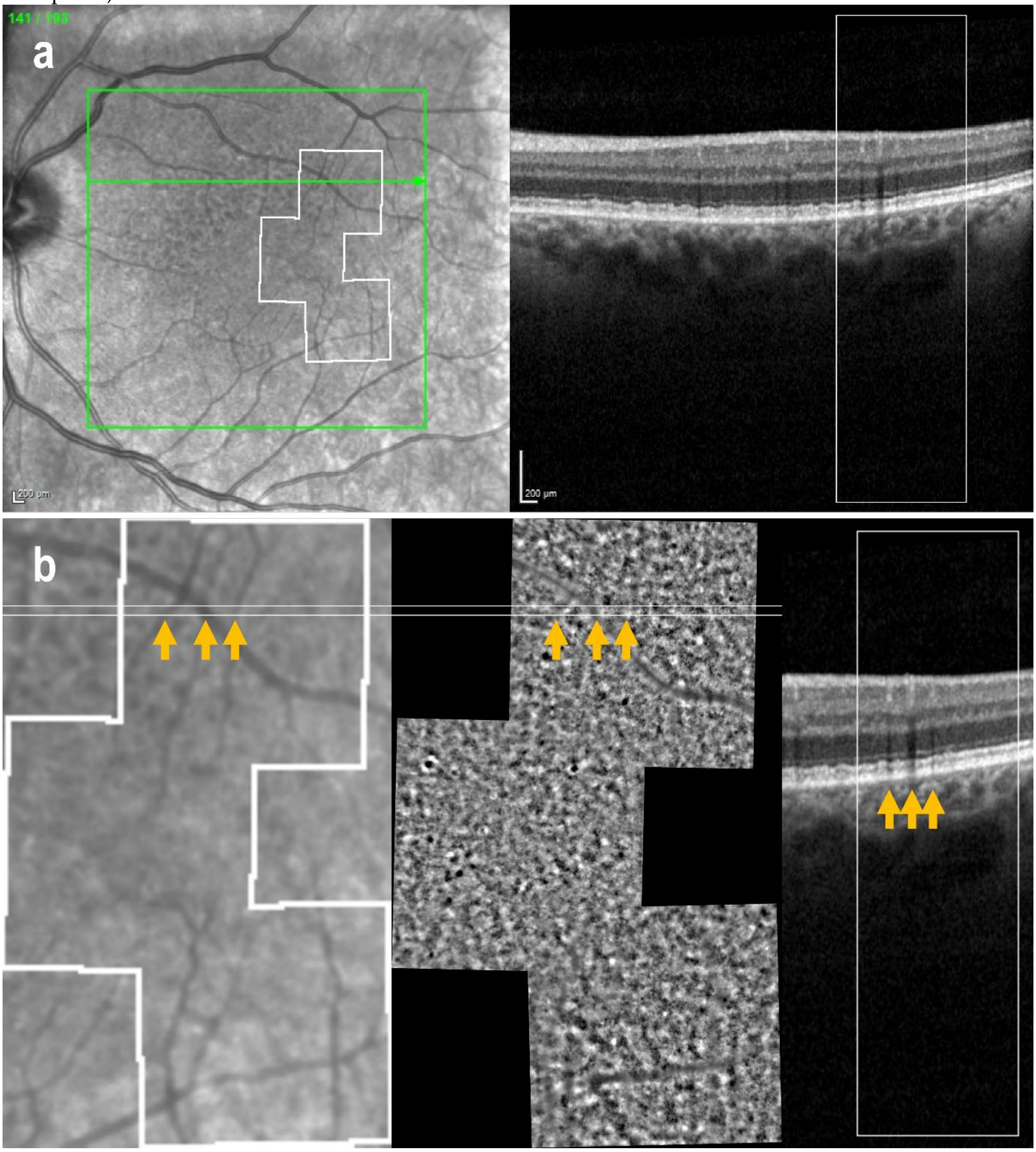

Figure S2. Subretinal drusenoid deposits in early AMD (Nasal quadrant of the left eye, female, 72 years). The infrared (IR) fundus image **(a)** corresponds to the AO-TFI image **(b)**. The colored lines indicate the locations of the OCT B-scans showing stage 1 SDD **(c, pink line)**, stage 2 SDD **(d, yellow line)**, and stage 3 SDD **(e, blue line)**. Early SDD stages 1 and 2 on OCT sections **(c-d, white arrows)** are not well identified on the AO-TFI image **(b, white arrows)**. In contrast, AO-TFI reveals hyperreflective spots **(b, blue arrows)** where OCT scan shows advanced stage 3 SDD disrupting the ellipsoid zone **(e, blue arrows)**. The images were extracted from stack images 45, 60 and 70 of the full "Correlation fundus-AO-TFI-OCT" dataset available in Movie S5.

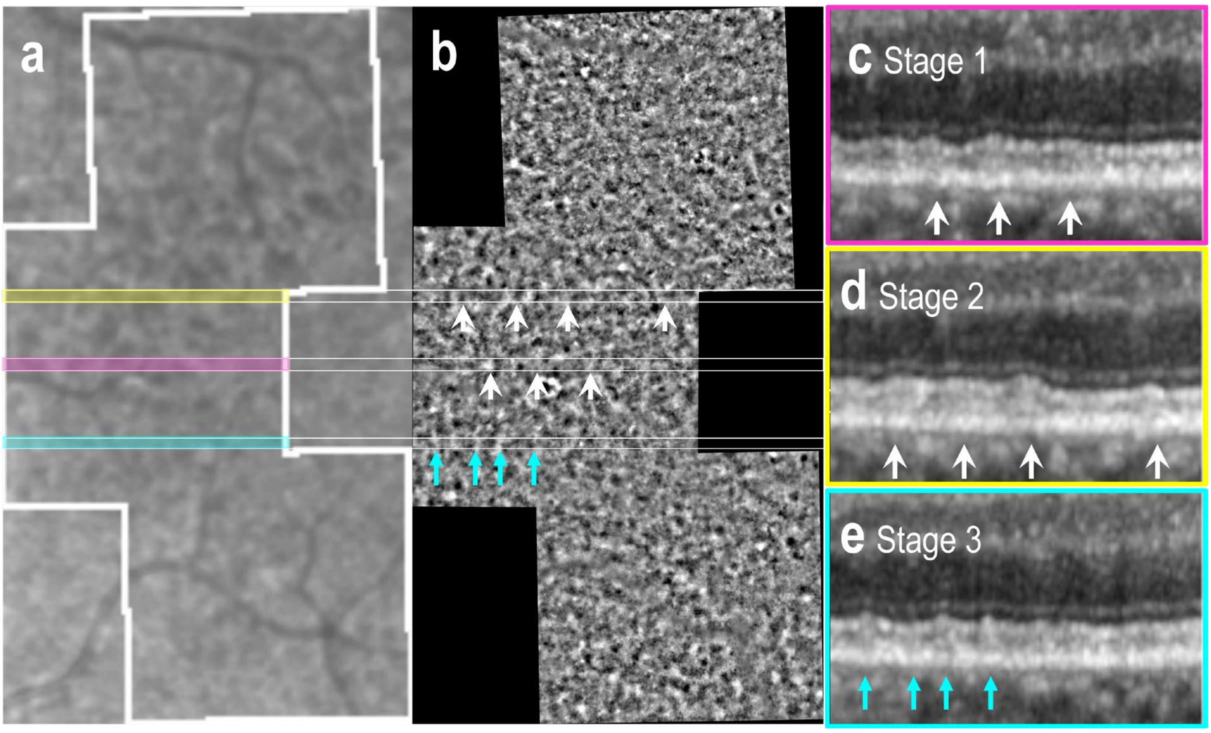

Supplement: Supplementary file 1 [file cells-14-00633-s001.zip › Cells-3592216_Supplementary-fig.pdf]
