# Supplementary material for: Adaptive Optics-Transscleral Flood Illumination Imaging of Retinal Pigment Epithelium in Dry Age-Related Macular Degeneration
Source: Cells. 2025 Apr 24;14(9):633. doi: 10.3390/cells14090633 (PMC12071642; doi:10.3390/cells14090633)
Supplement: Supplementary file 1 [file cells-14-00633-s001.zip › Cells-3592216_Supplementary-movies_legends.pdf]

Description of the supplementary movies

Supplementary Movie S1. Full stack of 120 "Correlation-fundus-AO-TFI-OCT" images of a left eye with early AMD and subretinal drusenoid deposits (Female, 72 years). Each image shows the AO-TFI mosaic (center) with the IR fundus cropped to fit the area imaged with AO-TFI (left panel) and the corresponding OCT B-scan (right panel).

Supplementary Movie S2. Full stack of 125 images "Correlation-fundus-AO-TFI-OCT" of a right eye with early AMD (Female, 67 years). Each image shows the AO-TFI mosaic (center) with the IR fundus cropped to fit the area imaged with AO-TFI (left panel) and the corresponding OCT B-scan (right panel).

Supplementary Movie S3. Full stack of 129 "Correlation-fundus-AO-TFI-OCT" images of a right eye with cuticular drusen in early AMD (Female, 67 years). Each image shows the AO-TFI mosaic (center) with the IR fundus cropped to fit the area imaged with AO-TFI (left panel) and the corresponding OCT B-scan (right panel).

Supplementary Movie S4. Full stack of 127 images "Correlation-fundus-AO-TFI-OCT" of a right eye with soft drusen in intermediate AMD (Female, 62 years). Each image shows the AO-TFI mosaic (center) with the IR fundus cropped to fit the area imaged with AO-TFI (left panel) and the corresponding OCT B-scan (right panel).

Supplementary Movie S5. Full stack of 119 images "Correlation-fundus-AO-TFI-OCT" of a left eye with subretinal drusenoid deposit in early AMD (Female, 72 years). Each image shows the AO-TFI mosaic (center) with the IR fundus cropped to fit the area imaged with AO-TFI (left panel) and the corresponding OCT B-scan (right panel).

Supplementary Movie S6. Full stack of 110 images "Correlation-fundus-AO-TFI-OCT" of a left eye with geographic atrophy and advanced reticular pseudodrusen (Female, 72 years). Each image shows the AO-TFI mosaic (center) with the IR fundus cropped to fit the area imaged with AO-TFI (left panel) and the corresponding OCT B-scan (right panel).

Supplementary Movie S7. Full stack of 119 images "Correlation-fundus-AO-TFI-OCT" of a right eye with atrophic AMD (Female, 72 years). Each image shows the AO-TFI mosaic (center) with the IR fundus cropped to fit the area imaged with AO-TFI (left panel) and the corresponding OCT B-scan (right panel). Stack images 37 and 61 were used to illustrate the Figure 5.

Supplementary Movie S8. Full stack of 125 images "Correlation-fundus-AO-TFI-OCT" of a left eye with atrophic AMD (Female, 80 years). Each image shows the AO-TFI mosaic (center) with the IR fundus cropped to fit the area imaged with AO-TFI (left panel) and the corresponding OCT B-scan (right panel).
