# Supplementary figures and images for: Adaptive Optics-Transscleral Flood Illumination Imaging of Retinal Pigment Epithelium in Dry Age-Related Macular Degeneration
Source: Cells. 2025 Apr 24;14(9):633. doi: 10.3390/cells14090633 (PMC12071642; doi:10.3390/cells14090633)

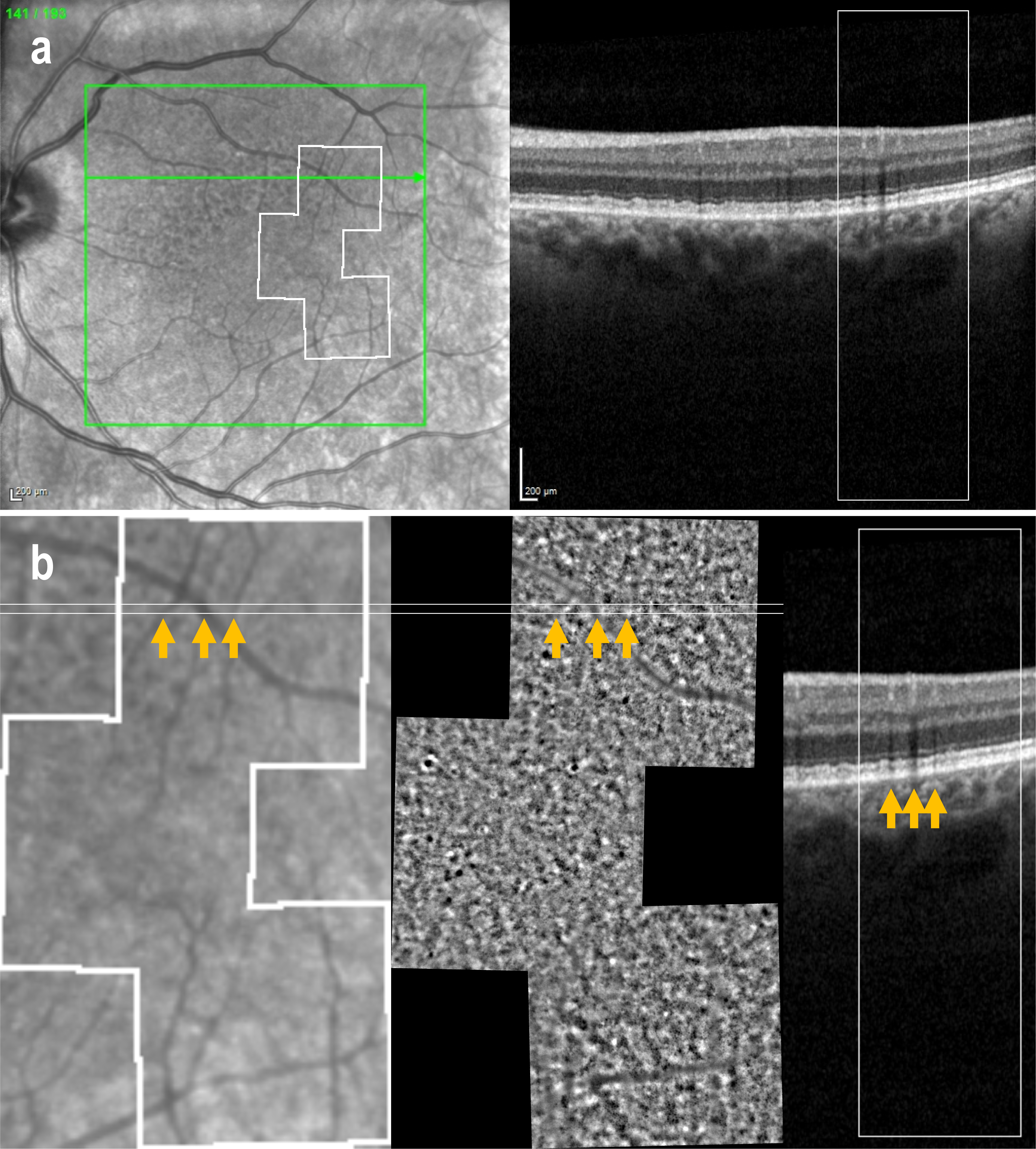

Supplement: Supplementary file 1 [file cells-14-00633-s001.zip › AO-TFI_AMD_FigS1.png]

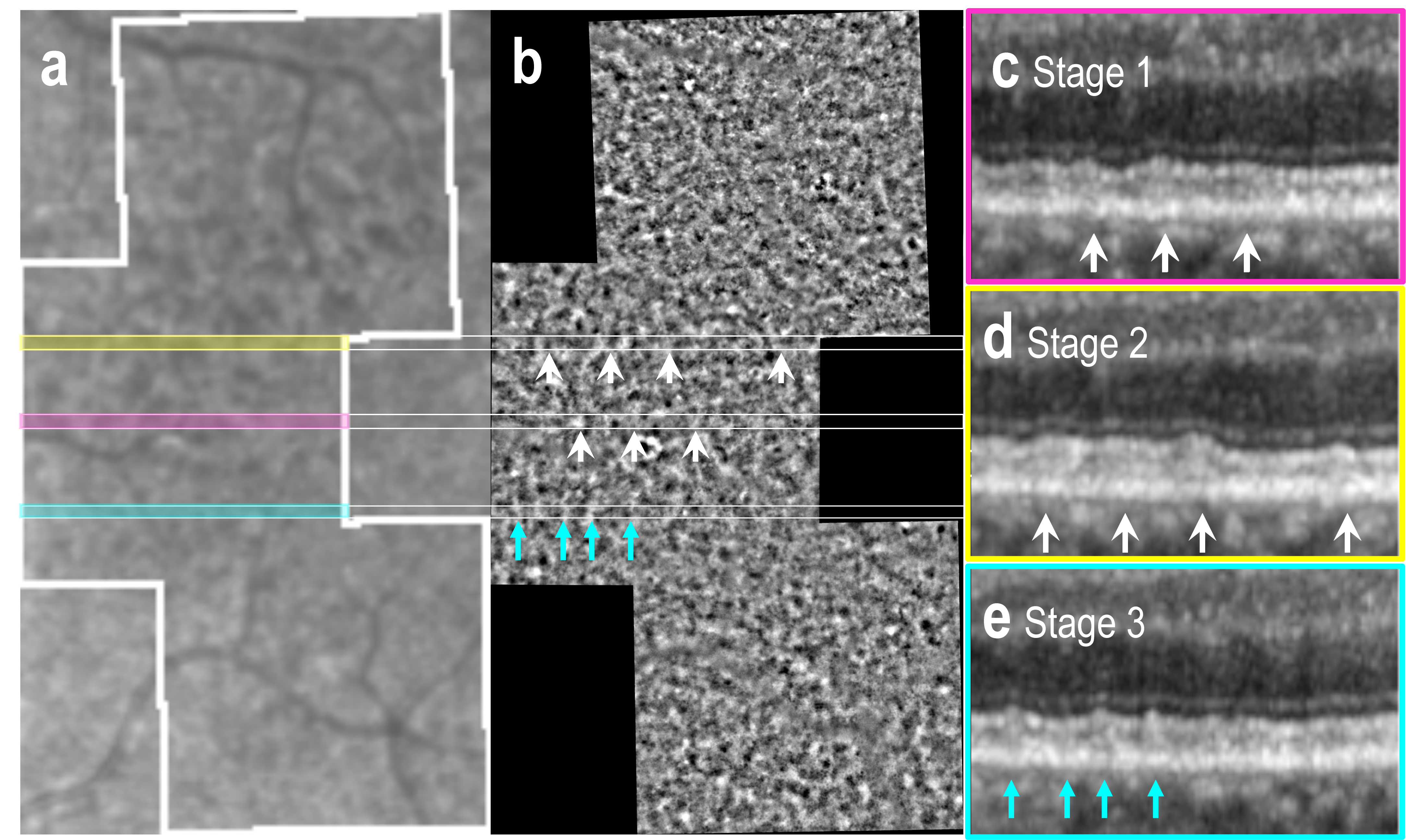

Supplement: Supplementary file 1 [file cells-14-00633-s001.zip › AO-TFI_AMD_FigS2.png]
